# Color Assessment of Feldspathic Ceramic with Two Different Thicknesses, Using Multiple Polymeric Cements

**DOI:** 10.3390/polym15020397

**Published:** 2023-01-12

**Authors:** Catarina Gomes, Francisco Martins, José Alexandre Reis, Paulo Durão Maurício, María Piedad Ramírez-Fernández

**Affiliations:** 1 Health Sciences PhD Program, Universidad Católica de Murcia UCAM, Campus de los Jerónimos nº135, 30107 Guadalupe, Murcia, Spain; 2 Oral Rehabilitation Department, Instituto Universitário Egas Moniz, Quinta da Granja—Monte de Caparica, 2829-511 Almada, Portugal; 3 CiiEM, Instituto Universitário Egas Moniz, Quinta da Granja, Monte da Caparica, 2829-511 Almada, Portugal

**Keywords:** feldspathic ceramics, resin cement, flowable resin, color, thickness

## Abstract

The purpose of this study was to evaluate the color changes of feldspathic ceramics CEREC Blocs (Dentsply Sirona, Milford, DE, USA) when cemented with different luting agents, while varying the ceramic thickness. Seventy ceramic discs of feldspathic ceramic (A2 shade) were obtained with 0.5 and 0.8 mm thicknesses. Seventy composite discs (A3 shade) 1 mm in thickness were used as substrates. After being polished and conditioned, the ceramic and composite discs were cemented with different resin cements and a flowable composite: Variolink^®^ Esthetic Light, Neutral and Warm (Ivoclar Vivadent, Schaan, Liechtenstein); RelyX^TM^ Veneer B0.5, Translucent and A3 Opaque/yellow shades (3M Oral Care, St. Paul, MN, USA); G-aenial^®^ Universal Flow A2 (GC Europe, Leuven, Belgium). Color difference (ΔE) was determined using a spectrophotometer. A two-way ANOVA and multiple comparisons were performed using the Bonferroni method with a 95% confidence interval. Variolink^®^ Neutral showed the highest ΔE (15.12 ± 0.71) and RelyX^TM^ Veneer A3 the lowest value (1.59 ± 0.33). There are no statistically significant differences between the two ceramic thicknesses for Variolink^®^ Light (*p* = 0.230) and RelyX^TM^ Veneer B0.5 (*p* = 0.318) cements. The feldspathic ceramic final color is influenced by the cement used and the ceramic thickness. The use of different cements in a thin ceramic has a clinically significant impact on the final esthetic result.

## 1. Introduction

Teeth color is a phenomenon determined by the sum of its primary and secondary optical properties. This phenomenon is influenced by several factors, such as the light source, brightness, opacity and visual perception of the observer [1]. Combining the optical properties of natural teeth with different restorative materials has become an esthetic challenge in dentistry [2].

Dental ceramics have been widely used in anterior esthetic restorations due to their biocompatibility and mechanical and optical properties [3,4]. However, the shade of the underlying tooth, the restoration core, the ceramic material itself and the cement used appear to affect the final shade of the restoration and its long-term clinical success [5,6,7].

Dental ceramics can be divided into glass ceramics, polycrystalline ceramics and resin matrix ceramics [8,9]. Feldspathic ceramics are essentially made up of kaolin, potassium feldspar and quartz (silica). Their quartz percentage is approximately 15% and contributes to the translucency and crystalline phase [8,10,11]. They have excellent esthetic properties and are mostly used for veneers, inlays, onlays, all-ceramic crowns and as a coating for metal-ceramic crowns [11,12].

Traditionally, feldspathic ceramics were obtained through a layering technique that evolved into pressing techniques and CAD/CAM (computer aided design/computer aided manufacturing). In this way, it became possible to create indirect restorations from ceramic blocks, simplifying conventional manufacturing procedures, guaranteeing long durability, fitness and esthetics to the restorations [13]. However, during the feldspathic ceramics’ CAD/CAM manufacturing stages, color differences may be detectable, since when they are produced very thin, these ceramics will have high translucency, generating a ceramic final color contrast relative to the initial ceramic block [14].

As always, the major challenge is to achieve the optical properties of natural teeth using synthetic materials. Among these materials, ceramics have an optical behavior quite similar to natural teeth and try, as much as possible, to reproduce their esthetic appearance [15,16].

Generally, stronger ceramic systems have greater opacity and less favorable esthetics due to their high crystalline content. On the other hand, more translucent ceramic systems allow greater light transmission through the material, providing a more natural appearance to the restoration. However, translucency makes the color matching process more complex [6].

The thickness, type of material and combination of ceramic layers also influence the final color of the restoration. Different thicknesses of ceramic can be used, depending on the type of restoration intended. To improve their esthetic result, it is important to evaluate the effect that the material thickness has on the optical properties of the restoration [17,18].

Previous studies have shown that changes in ceramic thickness can drastically influence the restoration final shade [17,18,19,20]. A thin ceramic layer has a reduced masking ability, revealing the color of the underlaying tooth structure and cement used [21]. The masking ability is defined as the capacity to mask a colored background [22]. Hiding a substrate with resin cements can be challenging because the cement layers are thin. However, the substrate’s color cannot be changed; it can only be masked [22,23].

Therefore, when using a ceramic restoration with high translucency and a dark background, it is important to carefully consider the choice of cement in order to effectively mask the underlying substrate [4,24,25].

Resin cements are polymers that are designed to provide strong, stable and durable bonds between indirect restorations and teeth [26]. These cements have several benefits, including good esthetics, low solubility in oral fluids, good mechanical properties and a strong interaction with tooth structure, which leads to favorable clinical outcomes [6,7,26,27].

The final color of resin cement also depends on the materials it is composed of. Most resin cements consist of an organic resin matrix, a coupling agent and an inorganic filler [23,28]. The resin matrix is typically made up of dimethacrylate monomers such as bisphenol-A-ethoxy dimethacrylate (BisEMA), dimethacrylate monomers (BisGMA), urethane dimethacrylate (UDMA), triethylene glycol dimethacrylate (TEGDMA) and 2-hydroxyethyl methacrylate (HEMA) [23,29].

The monomers that make up the resin matrix and the inorganic fillers have different refractive indices, which can influence the optical properties of the material, such as its translucency, due to the refraction and reflection at the material interfaces [30,31].

In addition to the influence of the refractive index, several studies have been developed in order to understand the influences that the size, particle type and constituents of the fillers have on the appearance of the esthetic restoration [28,32,33,34].

Therefore, when bonding a ceramic restoration, the selection of the proper type and color of cement is a critical factor in order to achieve an ideal esthetic result. The color stability of the cement under the restoration is also essential for its long-term clinical success [7,35].

When the light hits a cemented restoration in the oral cavity, different phenomena can occur. It can be transmitted through the different layers of the restoration, reflected from each of them, and refracted through their limits/edges, which can lead to variations in the color perception of the restoration by the observer [4].

Color determination can be performed using both visual and instrumental methods. The devices used in the instrumental measurement method aim to increase the accuracy of the color combination, its standardization and its numerical expression. However, this method should be complemented, whenever possible, with the visual method, leading to more predictable esthetic results [5,36]. The instrumental method of color measurement includes spectrophotometers, colorimeters and intraoral digital scanners [36]. More recently, digital photography has also been considered as a method of evaluating color [37].

Material color changes after different procedures can be calculated through their color differences (ΔE). The color difference (ΔE) indicates whether the change in color is perceptible to the human eye, and its limit value is not consensual in the literature. Several studies indicate that values of color difference (ΔE) greater than 3.5 between restorative material and natural teeth are not considered esthetically acceptable [5].

The color parameters can be quantified using the color ordering system developed by the Commission Internationale de l’Eclairage (CIELAB) in 1976. This system allows the representation of color three-dimensionally through three coordinates: L*, which represents the luminosity, ranging from 0 (black) to 100 (white); a*, which quantifies the red (positive value) and green (negative value); and b*, which quantifies the yellow (positive value) or blue (value negative) [38,39].

The spectrophotometer can reveal small color differences not detectable by the human eye. This system measures the reflection and transmission curves of the observed object, providing the spectral curve; it is limited to the measurement of color in the visible spectrum range [39]. Spectrophotometers have been considered the most useful, accurate and applicable systems for esthetic restorations, resembling natural teeth [40].

The aim of this in vitro study was to investigate the effects of feldspathic ceramic thickness and resin cements on the final esthetic outcome of ceramic restorations. The null hypothesis was that ceramic thickness and resin cement shade would not affect the color of a feldspathic ceramic.

## 2. Materials and Methods

CAD-CAM feldspathic ceramic (CEREC Blocs; Dentsply Sirona, Milford, DE, USA) (Table 1) blocks, shade A2, were cut perpendicularly into seventy samples, with 0.5 mm (*n* = 35) and 0.8 mm (*n* = 35) thicknesses and a 12 mm diameter, using a water-cooled diamond saw (Isomet 1000; Buehler, Lake Bluff, IL, USA) at a speed of 450 rpm, cooled with deionized water. It was decided to dispense with the first and last cuts of each ceramic block to standardize the samples.

All samples were polished with a grinding machine (LabolPol-4; Stuers, Cleveland, OH, USA) with sequential grinding papers (Carbimet 2; Buehler, Lake Bluff, IL, USA) of ISO/FEPA 400, 600 and 1200 grits at a constant speed of 100 rpm, for 15 s each.

Seventy composite resin disks (Filtek^TM^ Supreme XTE A3 Body Shade; 3M ESPE, St. Paul, MN, USA) (Table 1), with a diameter of 12 mm and 1 mm thickness, were obtained through a resin former (Porcelain Sampler, Ref. 7015, Smile Line, Saint-Imier, Switzerland) and light-cured for 40 s using a LED unit (Elipar^TM^; 3M, St. Paul, MN, USA) at high intensity (1000 mW/cm^2^) according to the manufacturer’s instructions.

A digital caliper was used to check all ceramic and resin samples at three different points, in order to ensure the correct thickness in all samples.

**Figure 1:**
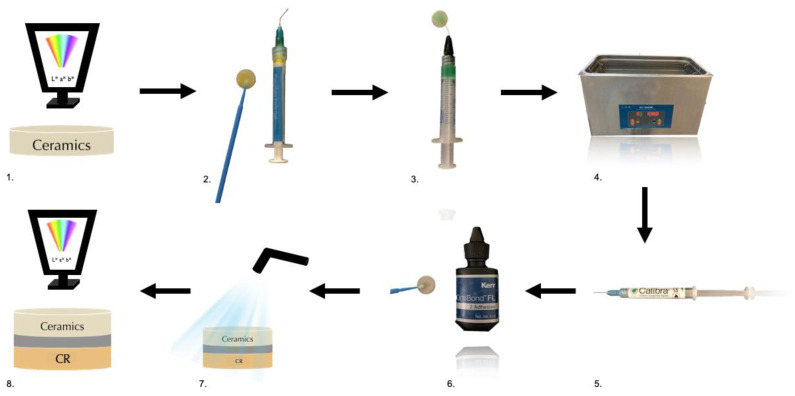
Diagram of the study design. (**1**) Ceramic L*a*b measurement; (**2**) 9.6% hydrofluoridric acid; (**3**) 37% orthophosphoric acid; (**4**) ultrasonic bath; (**5**) silane; (**6**) adhesive; (**7**) ceramic luting with different cements; (**8**) cemented ceramic L*a*b measurement.

To start the preparation of the ceramic surface, 9.6% hydrofluoric acid (PulpDent Corporation, Watertown, MA, USA) was applied for 90 s. Then it was rinsed with distilled water for 60 s and air dried, followed by the application of 37% orthophosphoric acid (R&S, France) (Figure 1).

The ceramic samples were then cleansed for 4 min in an ultrasonic bath with distilled water. To ensure dryness, the samples were removed from the ultrasonic bath and flushed with 96% alcohol for 30 s.

A silane coupling agent (Calibra; Dentsply Sirona, Milford, DE, USA) was applied for 20 s with a microbrush and allowed to evaporate for 60 s. Finally, an adhesive (Optibond^TM^ FL; Kerr, Scafati, Italy) was applied without curing (Figure 1).

Ceramic and resin disks were randomly paired using Microsoft Excel’s (Microsoft Corporation, Washington, DC, USA) RAND() formula and assigned to the following experimental groups according to the resin-based luting agent (*n* = 5): Variolink^®^ Esthetic Light, Neutral and Warm (Ivoclar Vivadent, Schaan, Liechtenstein); RelyX^TM^ Veneer B0.5, Translucent and A3 Opaque/yellow shades (3M Oral Care, St. Paul, MN, USA); G-aenial^®^ Universal Flow A2 (GC Europe, Leuven, Belgium).

Cementation was performed by exerting a constant pressure of 20N for 60 s, using a weight of 2 kg and a glass plate to standardize the luting agent thickness, followed by light curing (1000 mw/cm^2^) with an Elipar^TM^ (3M, St. Paul, MN, USA) for 60 s.

After this procedure, the samples were placed in a dry environment, at room temperature and in the absence of light for 24 h.

Color was determined according to the CIELAB color scale relative to the standard illuminant D65 on a reflection spectrophotometer (Spectro Shade, MHT S.p.A., Milan, Italy), allowing the object’s transmittance and spectral reflectance measurement under standardized conditions. The spectrophotometer was calibrated according to the manufacturer’s instructions, and color measurement was performed at the center of each sample for each ceramic sample, before and after its cementation on a gray background [41,42].

Color difference (ΔE) was determined by L*, a* and b* values obtained by a spectrophotometer above a grey background before and after cementation (Figure 1). The following formula was applied to calculate the color difference (ΔE) [42]:

(1) $$∆E=\sqrt{{\left(∆L^{*}\right)}^{2}+{\left(∆a^{*}\right)}^{2}+{\left(∆b^{*}\right)}^{2}}$$

The normal distribution was verified using the Shapiro–Wilk test and the homogeneity of variances according to the Levene’s test. A two-way ANOVA was performed to evaluate the effects of the resin-based luting agents and the thickness of feldspathic ceramic (0.5 or 0.8 mm) on the ΔE measures. Post hoc comparisons were performed using the Bonferroni method with a 95% confidence interval. All statistical tests were performed with a statistical software program (IBM SPSS v27; IBM Corp., New York, NY, USA) (α = 0.05).

## 3. Results

**Figure 2:**
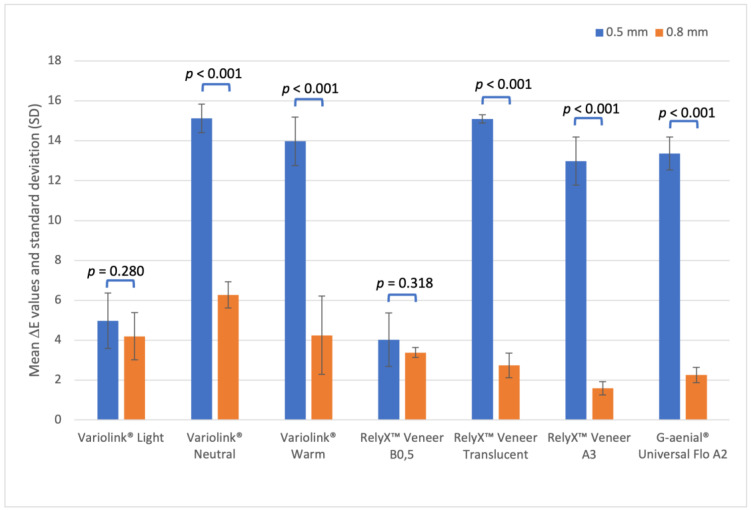
Mean ΔE values and standard deviation (SD) bars obtained for bonded ceramic samples according to the resin-based luting agent and the feldspathic ceramic thickness.

Table 2 and Figure 2 report the mean values and standard deviations for each resin-based material and for 0.5 mm and 0.8 mm ceramic thicknesses. The mean ΔE results (Table 2) reveal that Variolink^®^ Neutral (15.12 ± 0.71) and RelyX^TM^ Veneer Translucent (15.10 ± 0.21) had the highest color variation regarding the 0.5 mm ceramic, and Variolink^®^ Neutral (6.27 ± 0.66) for the 0.8 mm thickness. The lowest color variation on the thinnest ceramic was found with RelyX^TM^ Veneer B0.5 (4.03 ± 1.34), and for the thickest, with RelyX^TM^ Veneer A3 (1.59 ± 0.33).

For both thicknesses, the cements presented a statistically different ΔE values between them, with some exceptions shown in the Table 2.

Considering 0.5 mm ceramic thickness, the following cement groups did not show statistically significant differences (*p* > 0.05) (Table 2):

- Variolink^®^ Light and RelyX^TM^ Veneer B0.5;
- Variolink^®^ Neutral, Variolink^®^ Warm, RelyX^TM^ Veneer Translucent and G-aenial^®^ Universal Flo A2;
- Variolink^®^ Warm and RelyX^TM^ Veneer A3;
- RelyX^TM^ Veneer A3 and G-aenial^®^ Universal Flo A2.

Considering 0.8 mm ceramic thickness, the following cement groups did not show statistically significant differences (*p* > 0.05) (Table 1):

- Variolink^®^ Light, Variolink^®^ Warm, RelyX^TM^ Veneer B0.5, RelyX^TM^ Veneer Translucent and G-aenial^®^ Universal Flo A2;
- Variolink^®^ Neutral and Variolink^®^ Warm;
- RelyX^TM^ Veneer B0.5 and RelyX^TM^ Veneer A3;
- RelyX^TM^ Veneer Translucent, RelyX^TM^ Veneer A3 and G-aenial^®^ Universal Flo A2.

All cements present a statistically significant difference (*p* < 0.001) (Table 2 and Figure 2) when ΔE results are compared between the ceramic thicknesses, apart from Variolink^®^ *Light* (*p* = 0.230) and RelyX^TM^ Veneer B0.5 (*p* = 0.318) for both thicknesses.

## 4. Discussion

Feldspathic ceramics are chosen not only due to their optical and esthetic properties, but also as their biomimicry of natural tooth structure [8,10,12,14,43]. The feldspathic ceramic translucency becomes a challenge for color management, especially when using different cements and ceramic thicknesses [21,44]. Resin cements have high stability and predictable results due to their good esthetics, clinical applicability and mechanical properties [6,45,46,47]. According to Hernandes et al. [48], different cement thicknesses lead to significant changes in the optical properties of the ceramic material, making important to standardize the cement thickness using a 20N force exerted by a weight of 2 kg, as shown in the study of Hoorizad et al. [49].

Ceramic thickness is an important factor that might influence the final color result. When using a thinner ceramic, the fraction of light that is not reflected penetrates its surface, where it is mostly transmitted, and as the ceramic thickness increases, the translucency decreases [25,50]. Therefore, we used different ceramic thicknesses of 0.5 mm [2,51,52,53] and 0.8 mm [24,54,55]. Composite discs 1 mm in thickness were used as a substrate to standardize the background and create a common clinical situation [27,47,56].

Several methods are described in the literature to evaluate the color differences between samples. However, a spectrophotometer has been considered a precise method and is clinically available [36,41,49,55,57,58]. The spectrophotometer CIELab system data were obtained and analyzed through ΔE calculation formula to compare the color differences between samples [41,49,55,58,59,60,61,62].

The ΔE reference value is widely discussed among peers, and there is no determined or accepted value [39,63]. Douglas et al. [64] stated that for 50% of observers, the mean color perceptibility tolerance was a 2.6 ΔE. However, the acceptability tolerance was wider; the ΔE mean value was 5.5. Later Da Silva et al. [63] referred to 2.69 ΔE as the accepted value, and Chen et al. [35], a lower value of 2.0 ΔE. In the present study, ΔE ≤ 3.3 was considered as the mean reference value [49,55,65,66,67].

For both thicknesses, we found statistically significant differences (Table 2) between most of the tested cements (*p* < 0.001). This suggests that there is a clinically visible color change when varying the cement, as Xing et al. [67], Pires et al. [47], Dede et al. [68], Pissaia et al. [27], Czigola et al. [69], Gugelmin et al. [54], Hoorizad et al. [49] and Carrabba et al. [41] stated. In a previous study conducted by our team, Gomes et al. [70] concluded, like Tabatabaei et al. [71], that there is a clinically detectable difference between cements.

Within tested cements, there were statistically significant differences between the two ceramic thicknesses, excluding Variolink^®^ Light and RelyX^TM^ Veneer B0.5 cements. Both these cements are similar in shade to the ceramic color, and thus, for each of them, the ΔE value is small.

Furthermore, these results suggest that the ceramic’s thickness variation influences the final color of the restoration. These clinical detectable differences are in agreement with Xing et al. [67], Pires et al. [47], Igiel et al. [21], Czigola et al. [69], Tamam et al. [44], Carrabba et al. [41] and Gomes et al. [70].

The ΔE mean values decreased while the ceramic thickness increased, as reported by Tomaselli et al. [72] and Igiel et al. [21]. The greater the ceramic thickness, the greater the ability to mask and improve the final color, since the light reflection depends more on the ceramic than on the cement or substrate [47,73].

As shown in Figure 2, the color difference (ΔE) was not noticeable for the thickest ceramic when cemented with RelyX^TM^ Veneer Translucent, RelyX^TM^ Veneer A3, or G-aenial^®^ Universal Flo A2, which was expected because a thick ceramic is able to mask the cement and the tooth core. Nonetheless, color differences were clinically visible for all other cements, most likely due to their pigment content and shade color.

The greatest color variation occurred in the thinner ceramic, which has higher translucency, and therefore, a lower capacity to conceal the substrate. In this case, the cement plays a crucial role in masking the color of the substrate.

The cements tested have different brands and shades that are marketed to appeal to clinicians. White shades are often labeled as light or B0.5; neutral shades are labeled as neutral or translucent; and yellower shades are labeled as Warm, A3 or A2.

Relyx^TM^ Veneer Translucent and Variolink^®^ Neutral cements had large ΔE values, indicating higher translucency compared to the other tested cements.

Relyx^TM^ Veneer B0.5 and Variolink^®^ Light cements had less variation from the initial ceramic color, suggesting less translucency.

The remaining cements, Relyx^TM^ Veneer A3, Variolink^®^ Warm and G-aenial^®^ Universal Flo A2, showed similar behavior, including intermediate deviations (ΔE) from the initial ceramic color, suggesting that cements with higher concentrations of yellowish pigments have intermediate translucency.

Therefore, the varied results obtained for the different cements can be explained through the differences in their chemical compositions and their effects on the optical properties of dental restorations.

Several studies have shown that the color and translucency of esthetic restorations are influenced by the compositions of the fillers and resin matrix, and the pigments and other chemical components present in each resin cement [32,33,74].

Pigments are substances that are added but can also be removed from the resin matrix of the cement to give it a specific color. In the case of white cement, a white pigment, such as titanium dioxide, may be used to achieve the desired shade [75]. The amount of pigment used can affect the intensity of the white color. More pigment results in a brighter shade, and less pigment results in a duller shade.

Other common pigments that change the yellow shade are hansa yellow and benzimidazolone orange. The amount of pigment used in resin dental cements can affect the color intensity: more pigment results in a darker shade and less pigment results in a lighter shade. The color of the cement can also be affected by the types and amounts of fillers used in the cement, as some fillers have a natural color that may show through the resin matrix [76].

Most likely, Relyx^TM^ Veneer A3, Variolink^®^ Warm and G-aenial^®^ Universal Flo A2 have more hansa yellow and benzimidazolone orange and less titanium dioxide; and Relyx^TM^ Veneer B0.5 and Variolink^®^ Light cements have more titanium dioxide and less hansa yellow and benzimidazolone orange.

The types and amounts of fillers used in resin dental cements can affect the color. Some fillers, such as glass or silica particles, have a natural color that may show through the resin matrix and affect the overall color of the cement. However, most fillers do not have a strong color and are generally not used to significantly affect the overall color of the cement [74]. However, it has not yet been studied in depth which fillers cause changes in color in terms of opalescence and translucency; and the differences between cement brands lead to heterogeneous results [28,34].

Nevertheless, we aimed in removing variables by limiting the composition of the material to only one type of ceramic and homogenizing the micromorphology of the material with surface treatment, thereby normalizing other optical behavior properties, such as light absorption, reflection, scattering and refraction of the material. Furthermore, our continuing research aims at evaluating this further.

## 5. Conclusions

Within the limitations of this in vitro study, it is possible to conclude that the thickness of feldspathic ceramic veneers can affect their final color.

Considering the studied ceramic thicknesses, the thicker ceramic veneer, which had lower translucency, resulted in a minor change in the final color of the restoration, making it esthetically more similar to the color of the initial ceramic.

It can also be concluded that the color of the cement and its ability to mask the color of the ceramic material underneath affect the final color of the restoration. Of the cements studied, the Relyx^TM^ Veneer B0.5 cement, had the smallest color change when used with a thinner veneer. However, when using a thicker ceramic material, the cement that showed the least color change was the Relyx^TM^ Veneer A3 cement.

Clinicians should consider materials that meet current esthetic needs. The choice of ceramic thickness and the color of the cementation material are crucial for achieving a good esthetic result. In a clinical setting, ceramic veneers may not always be 0.5 or 0.8 mm thick. They may be thinner or thicker, and it is important for the clinician to take special care to ensure the masking ability of the cement in order to meet esthetic requirements. Clinicians should be aware that if a non-translucent cement is used, the thinner part of the ceramic veneer near the gum line may appear lighter or more yellow in color compared to other parts of the veneer.

## Figures and Tables

**Table 1:** Manufacturers and compositions of ceramics, resin-based materials and luting agents studied.

Material and Manufacturer	Composition	Batch Number
Cerec^®^ Blocs C/PC VITA Shade: A2 CAD-CAM feldspathic ceramic	SiO_2_ (56–64%), Al_2_O_3_ (20–23%), Na_2_O (6–9%), K_2_ (6–8%), CaO (0.3–0.8%), TiO2 (0.0–0.1%), pigments < 0.1%.	66301
Filtek Supreme XTE 3M Oral Care Shade: A3 Body Nanofilled composite resin	UDMA, Bis-GMA, Bis-EMA, Silica (20 nm) Zirconia (4–10 nm). Size of the particles together 0.6 to 10 μm. Inorganic particles represent 72.5% of the total charge.	N859611
Variolink Esthetic LC Ivoclar VivadentShade: Light, Neutral and WarmResin cement	Urethane dimethacrylate, methacrylate monomers, inorganic fillers Ytterbium trifluoride and spheroid oxide mixed. Primers, stabilizers and pigments. Particle size is from 0.04 to 0.2 μm. Inorganic charge is approximately 38%.	v48653 w05218 w06171
RelyX Veneer 3M Oral Care Shade: B0.5, A3 and Translucent Resin cement	Bis-GMA, TEGDMA, Zirconia/silica, modified silica. Particle loading approximately 66% by weight, particle size approximately 0.6 mm, photoinitiator, pigments.	N862421 N816236 N843828
G-aenial Universal GC Corporation Shade: Flo A2 Flowable composite resin	Urethanedimetrylate, Bis-MEPP, TEGDMA (31%). Silicon dioxide (16 nm), Strontium glass (200 nm), pigments (69%), photoinitiator.	161202A

**Table 2:** Mean ΔE values and standard deviation (SD) between samples of cemented ceramics and the initial ceramic samples. Different letters in the same column indicate significantly different mean ΔE values.

Resin-Based Material	Ceramic Thickness		0.5 mm vs. 0.8 mm (*p*-Value)
	0.5 mm Mean ± SD	0.8 mm Mean ± SD	
Variolink^®^ Light	4.98 ± 1.39 ^A^	4.20 ± 1.18 ^A^	0.230 ^(a)^
Variolink^®^ Neutral	15.12 ± 0.71 ^B^	6.27 ± 0.66 ^B^	<0.001 ^(a)(*)^
Variolink^®^ Warm	13.97 ± 1.22 _BC_	4.25 ± 1.97 ^AB^	<0.001 ^(a)(*)^
RelyX^TM^ Veneer B0.5	4.03 ± 1.34 ^A^	3.38 ± 0.25 ^AC^	0.318 ^(a)^
RelyX^TM^ Veneer Translucent	15.10 ± 0.21 ^B^	2.74 ± 0.62 ^AD^	<0.001 ^(a)(*)^
RelyX^TM^ Veneer A3	12.98 ± 1.20 ^CD^	1.59 ± 0.33 ^CD^	<0.001 ^(a)(*)^
G-aenial^®^ Universal Flo A2	13.36 ± 0.82 ^BD^	2.26 ± 0.38 ^AD^	<0.001 ^(a)(*)^

(^a^) Two-way ANOVA and multiple comparisons (Bonferroni); (*) statistically significant difference for a 95% confidence interval.

## Data Availability

The data presented in this study are available on request from the corresponding author.

## References

[1] Joiner A. (2004). Tooth Colour: A Review of the Literature. J. Dent..

[2] Turgut S., Bagis B. (2013). Effect of Resin Cement and Ceramic Thickness on Final Color of Laminate Veneers: An In Vitro Study. J. Prosthet. Dent..

[3] Peumans M., van Meerbeek B., Lambrechts P., Vanherle G. (2000). Porcelain Veneers: A Review of the Literature. J. Dent..

[4] Tabatabaian F. (2019). Color Aspect of Monolithic Zirconia Restorations: A Review of the Literature. J. Prosthodont..

[5] Turgut S., Bagis B. (2011). Colour Stability of Laminate Veneers: An In Vitro Study. J. Dent..

[6] Dede D.Ö., Ceylan G., Yilmaz B. (2017). Effect of Brand and Shade of Resin Cements on the Final Color of Lithium Disilicate Ceramic. J. Prosthet. Dent..

[7] Kilinc E., Antonson S.A., Hardigan P.C., Kesercioglu A. (2011). Resin Cement Color Stability and Its Influence on the Final Shade of All-Ceramics. J. Dent..

[8] Gracis S., Thompson V., Ferencz J., Silva N., Bonfante E. (2016). A New Classification System for All-Ceramic and Ceramic-like Restorative Materials. Int. J. Prosthodont..

[9] Giordano R., McLaren E.A. (2010). Ceramics Overview: Classification by Microstructure and Processing Methods. Compend. Contin. Educ. Dent..

[10] Conrad H.J., Seong W.J., Pesun I.J. (2007). Current Ceramic Materials and Systems with Clinical Recommendations: A Systematic Review. J. Prosthet. Dent..

[11] Gomes E.A., Assunção W.G., Rocha E.P., Santos P.H. (2008). Cerâmicas Odontológicas: O Estado Atual. Cerâmica.

[12] Li R.W.K., Chow T.W., Matinlinna J.P. (2014). Ceramic Dental Biomaterials and CAD/CAM Technology: State of the Art. J. Prosthodont. Res..

[13] da Silva L.H., de Lima E., de Paula Miranda R.B., Favero S.S., Lohbauer U., Cesar P.F. (2017). Dental Ceramics: A Review of New Materials and Processing Methods. Braz. Oral Res..

[14] Sampaio C.S., Belfus J., Avila A., Cordero C., Freitte M., Ferrari V., Atria P.J., Jorquera G. (2021). Effect of Different Fabrication Steps on Color and Translucency of a CAD-CAM Feldspathic Ceramic. J. Esthet. Restor. Dent..

[15] Li Q., Yu H., Wang Y.N. (2009). Spectrophotometric Evaluation of the Optical Influence of Core Build-up Composites on All-Ceramic Materials. Dent. Mater..

[16] Soares C.J., Soares P.V., Pereira J.C., Fonseca R.B. (2005). Surface Treatment Protocols in the Cementation Process of Ceramic and Laboratory-Processed Composite Restorations: A Literature Review. J. Esthet. Restor. Dent..

[17] Dozić A., Kleverlaan C.J., Meegdes M., van der Zel J., Feilzer A.J. (2003). The Influence of Porcelain Layer Thickness on the Final Shade of Ceramic Restorations. J. Prosthet. Dent..

[18] Subaşı M.G., Alp G., Johnston W.M., Yilmaz B. (2018). Effect of Thickness on Optical Properties of Monolithic CAD-CAM Ceramics. J. Dent..

[19] Kürklü D., Azer S.S., Yilmaz B., Johnston W.M. (2013). Porcelain Thickness and Cement Shade Effects on the Colour and Translucency of Porcelain Veneering Materials. J. Dent..

[20] Blumentritt F.B., Cancian G., Saporiti J.M., de Holanda T.A., Barbon F.J., Boscato N. (2021). Influence of Feldspar Ceramic Thickness on the Properties of Resin Cements and Restorative Set. Eur. J. Oral Sci..

[21] Igiel C., Weyhrauch M., Mayer B., Scheller H., Lehmann K.M. (2018). Effects of Ceramic Layer Thickness, Cement Color, and Abutment Tooth Color on Color Reproduction of Feldspathic Veneers. Int. J. Esthet. Dent..

[22] Begum Z., Chheda P., Shruthi C.S., Sonika R. (2014). Effect of Ceramic Thickness and Luting Agent Shade on the Color Masking Ability of Laminate Veneers. J. Indian Prosthodont. Soc..

[23] Porojan L., Vasiliu R.D., Porojan S.D. (2022). Masking Abilities of Dental Cad/Cam Resin Composite Materials Related to Substrate and Luting Material. Polymers.

[24] Archegas L.R.P., Freire A., Vieira S., de Menezes Caldas D.B., Souza E.M. (2011). Colour Stability and Opacity of Resin Cements and Flowable Composites for Ceramic Veneer Luting after Accelerated Ageing. J. Dent..

[25] Chaiyabutr Y., Kois J.C., Lebeau D., Nunokawa G. (2011). Effect of Abutment Tooth Color, Cement Color, and Ceramic Thickness on the Resulting Optical Color of a CAD/CAM Glass-Ceramic Lithium Disilicate-Reinforced Crown. J. Prosthet. Dent..

[26] Braga R.R., Ballester R.Y., Ferracane J.L. (2005). Factors Involved in the Development of Polymerization Shrinkage Stress in Resin-Composites: A Systematic Review. Dent. Mater..

[27] Pissaia J.F., de Almeida Guanaes B.K., de Almeida Kintopp C.C., Correr G.M., da Cunha L.F., Gonzaga C.C. (2019). Color Stability of Ceramic Veneers as a Function of Resin Cement Curing Mode and Shade: 3-Year Follow-up. PLoS ONE.

[28] Kowalska A., Sokolowski J., Bociong K. (2021). The Photoinitiators Used in Resin Based Dental Composite—A Review and Future Perspectives. Polymers.

[29] de Oliveira D.C.R.S., Rocha M.G., Gatti A., Correr A.B., Ferracane J.L., Sinhoret M.A.C. (2015). Effect of Different Photoinitiators and Reducing Agents on Cure Efficiency and Color Stability of Resin-Based Composites Using Different LED Wavelengths. J. Dent..

[30] Miletic V., Jakovljevic N., Manojlovic D., Marjanovic J., Rosic A.A., Dramićanin M.D. (2017). Refractive Indices of Unfilled Resin Mixtures and Cured Composites Related to Color and Translucency of Conventional and Low-Shrinkage Composites. J. Biomed. Mater. Res. B Appl. Biomater..

[31] Ota M., Ando S., Endo H., Ogura Y., Miyazaki M., Hosoya Y. (2012). Influence of Refractive Index on Optical Parameters of Experimental Resin Composites. Acta Odontol. Scand..

[32] Shortall A.C., Palin W.M., Burtscher P. (2008). Refractive Index Mismatch and Monomer Reactivity Influence Composite Curing Depth. J. Dent. Res..

[33] Lim Y.-K., Lee Y.-K., Lim B.-S., Rhee S.-H., Yang H.-C. (2008). Influence of Filler Distribution on the Color Parameters of Experimental Resin Composites. Dent. Mater..

[34] Oivanen M., Keulemans F., Garoushi S., Vallittu P.K., Lassila L. (2021). The Effect of Refractive Index of Fillers and Polymer Matrix on Translucency and Color Matching of Dental Resin Composite. Biomater. Investig. Dent..

[35] Chang J., da Silva J.D., Sakai M., Kristiansen J., Ishikawa-Nagai S. (2009). The Optical Effect of Composite Luting Cement on All Ceramic Crowns. J. Dent..

[36] Liberato W.F., Barreto I.C., Costa P.P., de Almeida C.C., Pimentel W., Tiossi R. (2019). A Comparison between Visual, Intraoral Scanner, and Spectrophotometer Shade Matching: A Clinical Study. J. Prosthet. Dent..

[37] Sampaio C.S., Atria P.J., Hirata R., Jorquera G. (2019). Variability of Color Matching with Different Digital Photography Techniques and a Gray Reference Card. J. Prosthet. Dent..

[38] Ahn J.S., Lee Y.K. (2008). Color Distribution of a Shade Guide in the Value, Chroma, and Hue Scale. J. Prosthet. Dent..

[39] Vichi A., Louca C., Corciolani G., Ferrari M. (2011). Color Related to Ceramic and Zirconia Restorations: A Review. Dent. Mater..

[40] Malkondu O., Tinastepe N., Kazazoglu E. (2016). Influence of Type of Cement on the Color and Translucency of Monolithic Zirconia. J. Prosthet. Dent..

[41] Carrabba M., Vichi A., Tozzi G., Louca C., Ferrari M. (2022). Cement Opacity and Color as Influencing Factors on the Final Shade of Metal-Free Ceramic Restorations. J. Esthet. Restor. Dent..

[42] Bayindir F., Koseoglu M. (2020). The Effect of Restoration Thickness and Resin Cement Shade on the Color and Translucency of a High-Translucency Monolithic Zirconia. J. Prosthet. Dent..

[43] McLaren E.A., Figueira J. (2015). Updating Classifications of Ceramic Dental Materials: A Guide to Material Selection. Compend. Contin. Educ. Dent..

[44] Tamam E., Güngör M., Nemli S. (2020). How Are the Color Parameters of a CAD/CAM Feldspathic Ceramic of the Material Affected by Its Thickness, Shade, and Color of the Substructure?. Niger. J. Clin. Pract..

[45] Rodrigues R.B., de Lima E., Roscoe M.G., Soares C.J., Cesar P.F., Novais V.R. (2017). Influence of Resin Cements on Color Stability of Different Ceramic Systems. Braz. Dent. J..

[46] Almeida J.R., Schmitt G.U., Kaizer M.R., Boscato N., Moraes R.R. (2015). Resin-Based Luting Agents and Color Stability of Bonded Ceramic Veneers. J. Prosthet. Dent..

[47] Pires L.A., Novais P.M.R., Araújo V.D., Pegoraro L.F. (2017). Effects of the Type and Thickness of Ceramic, Substrate, and Cement on the Optical Color of a Lithium Disilicate Ceramic. J. Prosthet. Dent..

[48] Hernandes D.K.L., Arrais C.A.G., de Lima E., Cesar P.F., Rodrigues J.A. (2016). Influence of Resin Cement Shade on the Color and Translucency of Ceramic Veneers. J. Appl. Oral Sci..

[49] Hoorizad M., Valizadeh S., Heshmat H., Tabatabaei S.F., Shakeri T. (2021). Influence of Resin Cement on Color Stability of Ceramic Veneers: In Vitro Study. Biomater. Investig. Dent..

[50] Baldissara P., Wandscher V.F., Marchionatti A.M.E., Parisi C., Monaco C., Ciocca L. (2018). Translucency of IPS e.Max and Cubic Zirconia Monolithic Crowns. J. Prosthet. Dent..

[51] Kandil B.S.M., Hamdy A.M., Aboelfadl A.K., El-Anwar M.I. (2019). Effect of Ceramic Translucency and Luting Cement Shade on the Color Masking Ability of Laminate Veneers. Dent. Res. J..

[52] Mihali S.G., Lolos D., Popa G., Tudor A., Bratu D.C. (2022). Retrospective Long-Term Clinical Outcome of Feldspathic Ceramic Veneers. Materials.

[53] Tuzzolo Neto H., do Nascimento W.F., Erly L., Ribeiro R.A., de Sá Barbosa J., Zambrana J.M., Raimundo L.B., da Silva Mendes C., da Silva I.P., Mesquita A.M.M. (2018). Laminated Veneers with Stratified Feldspathic Ceramics. Case Rep. Dent..

[54] Gugelmin B.P., Miguel L.C.M., Baratto Filho F., da Cunha L.F., Correr G.M., Gonzaga C.C. (2020). Color Stability of Ceramic Veneers Luted with Resin Cements and Pre-Heated Composites: 12 Months Follow-up. Braz. Dent. J..

[55] Shadman N., Ebrahimi S.F., Shoul M.A., Kandi S.G., Rostami S. (2022). The Minimum Thickness of a Multilayer Ceramic Restoration Required for Masking Dark Background. Dent. Res. J..

[56] Lehmann K., Devigus A., Wentaschek S., Igiel C., Scheller H., Paravina R. (2017). Comparison of Visual Shade Matching and Electronic Color Measurement Device. Int. J. Esthet. Dent..

[57] Greţa D.C., Gasparik C., Colosi H.A., Dudea D. (2020). Color Matching of Full Ceramic Versus Metalceramic Crowns—A Spectrophotometric Study. Med. Pharm. Rep..

[58] Sonza Q.N., Della Bona A., Pecho O.E., Borba M. (2021). Effect of Substrate and Cement on the Final Color of Zirconia-Based All-Ceramic Crowns. J. Esthet. Restor. Dent..

[59] Khosravani S.R., Kahnamoui M.A., Kimyai S., Navimipour E.J., Mahounak F.S., Azar F.P. (2022). Final Colour of Ultratranslucent Multilayered Zirconia Veneers, Effect of Thickness, and Resin Cement Shade. Biomed. Res. Int..

[60] Comba A., Paolone G., Baldi A., Vichi A., Goracci C., Bertozzi G., Scotti N. (2022). Effects of Substrate and Cement Shade on the Translucency and Color of CAD/CAM Lithium-Disilicate and Zirconia Ceramic Materials. Polymers.

[61] Abreu J.L.B., Sampaio C.S., Benalcázar Jalkh E.B., Hirata R. (2021). Analysis of the Color Matching of Universal Resin Composites in Anterior Restorations. J. Esthet. Restor. Dent..

[62] Tabatabaian F., Khaledi Z., Namdari M. (2021). Effect of Ceramic Thickness and Cement Type on the Color Match of High-Translucency Monolithic Zirconia Restorations. Int. J. Prosthodont..

[63] Silva J.D., Park S.E., Weber H.-P., Ishikawa-Nagai S. (2008). Clinical Performance of a Newly Developed Spectrophotometric System on Tooth Color Reproduction. J. Prosthet. Dent..

[64] Douglas R.D., Steinhauer T.J., Wee A.G. (2007). Intraoral Determination of the Tolerance of Dentists for Perceptibility and Acceptability of Shade Mismatch. J. Prosthet. Dent..

[65] Kolbeck C., Rosentritt M., Lang R., Handel G. (2006). Discoloration of Facing and Restorative Composites by UV-Irradiation and Staining Food. Dent. Mater..

[66] Pissaia J.F., Correr G.M., Gonzaga C.C., da Cunha L.F. (2015). Influence of Shade, Curing Mode, and Aging on the Color Stability of Resin Cements. Braz. J. Oral Sci..

[67] Xing W., Chen X., Ren D., Zhan K., Wang Y. (2017). The Effect of Ceramic Thickness and Resin Cement Shades on the Color Matching of Ceramic Veneers in Discolored Teeth. Odontology.

[68] Dede D.Ö., Sahin O., Özdemir O.S., Yilmaz B., Celik E., Köroğlu A.S. (2017). Influence of the Color of Composite Resin Foundation and Luting Cement on the Final Color of Lithium Disilicate Ceramic Systems. J. Prosthet. Dent..

[69] Czigola A., Abram E., Kovacs Z.I., Marton K., Hermann P., Borbely J. (2019). Effects of Substrate, Ceramic Thickness, Translucency, and Cement Shade on the Color of CAD/CAM Lithium-Disilicate Crowns. J. Esthet. Restor. Dent..

[70] Gomes C., Martins F., Reis J.A., Albacete-Martinez C.P., Maurício P.D. (2022). Final Esthetic Result of Ceramic Restorations Cemented with Different Colors of Cement. Clin. Exp. Dent. Res..

[71] Tabatabaei M.H., Matinfard F., Omrani L.R., Mahounak F.S., Ahmadi E. (2019). Evaluation of the Final Color of Ceramic Veneers with Different Self-Adhesive Resin Cements. Open Dent. J..

[72] de Oliveira Tomaselli L., de Oliveira D.C.R.S., Favarão J., da Silva A.F., de Carvalho Panzeri Pires-de-Souza F., Geraldeli S., Sinhoreti M.A.C. (2019). Influence of Pre-Heating Regular Resin Composites and Flowable Composites on Luting Ceramic Veneers with Different Thicknesses. Braz. Dent. J..

[73] Tabatabaian F., Karimi M., Namdari M. (2020). Color Match of High Translucency Monolithic Zirconia Restorations with Different Thicknesses and Backgrounds. J. Esthet. Restor. Dent..

[74] Lee Y.-K. (2008). Influence of Filler on the Difference between the Transmitted and Reflected Colors of Experimental Resin Composites. Dent. Mater..

[75] Lee B.-S., Wang D.-M. (2015). Dental Bonding Agent and Coating Agent. U.S. Patent.

[76] da Silva Fontes A., di Mauro E., Antonia L.H.D. (2012). Study of the Influence of Pigments in the Polymerization and Mechanical Performance of Commercial Dental Composites. Rev. Odontol. Bras. Cent..

